# Quercetin selectively targets gingival squamous cell carcinoma through apoptosis induction, autophagy suppression, DNA damage, and chemosensitization

**DOI:** 10.3389/fonc.2026.1890978

**Published:** 2026-09-24

**Authors:** Abdelhabib Semlali, Sana Baroudi, Vijaykumar D. Nimbarte, Shreya S. Sonak, Hawraa Issa, Julie Cornillaut, Mikhlid H. Almutairi, Mahmoud Rouabhia

**Affiliations:** 1 GREB Research Group (Groupe de recherche en Écologie Buccale), Faculty of Dentistry, Laval University, Québec, QC, Canada; 2 Department of Pharmacy, Birla Institute of Technology and Science (BITS) Pilani, Telangana, India; 3 DREAM-CV Lab, Centre for Outcomes Research and Evaluation, Research Institute of the McGill University Health Centre, Montreal, QC, Canada; 4 Department of Zoology, College of Science, King Saud University, Riyadh, Saudi Arabia

**Keywords:** 5-fluorouracil, apoptosis, autophagy, chemosensitization, cisplatin, DNA damage, gingival squamous cell carcinoma, metastasis

## Abstract

**Background:**

Oral squamous cell carcinoma (OSCC) remains a major clinical challenge due to high recurrence rates, chemotherapy resistance, and severe treatment-related toxicity. Although quercetin, a naturally occurring flavonoid, has demonstrated anti-cancer properties, its selective activity and molecular mechanisms in distinct OSCC subtypes remain insufficiently characterized.

**Methods:**

In this study, we investigated the anti-tumor effects of quercetin in human gingival squamous cell carcinoma (Ca9-22), tongue carcinoma (SCC-9 and CAL-27), and non-malignant oral epithelial (GMSM-K) cells.

**Results:**

Quercetin (0–100 µM) selectively reduced the viability, proliferation, and clonogenic potential of gingival carcinoma cells while exerting limited cytotoxicity toward non-malignant oral epithelial cells, suggesting a favorable therapeutic index. Mechanistically, quercetin induced apoptosis through modulation of intrinsic and extrinsic apoptotic pathways, suppressed autophagy, reduced oxidative stress, promoted DNA damage, and inhibited migratory and metastatic potential. Transcriptomic profiling identified several apoptosis-related genes modulated by quercetin, including CASP9 and BIRC3, whose elevated expression was associated with improved overall survival in head and neck squamous cell carcinoma datasets. In addition, quercetin enhanced the anti-proliferative effects of cisplatin and 5-fluorouracil, supporting its potential role as a chemosensitizing agent in OSCC therapy. Molecular docking and molecular dynamics simulations further demonstrated stable interactions between quercetin and apoptosis- and DNA damage-related proteins, including CASP9, H2AX, and BIRC3.

**Conclusions:**

Collectively, these findings identify quercetin as a selective multi-target anti-cancer compound capable of modulating apoptosis, autophagy, oxidative stress, DNA damage, and metastatic pathways in gingival squamous cell carcinoma. These data support the translational potential of quercetin as a complementary strategy to enhance chemotherapy efficacy in oral cancer.

## 1 Introduction

Oral cancer (OC), predominantly represented by oral squamous cell carcinoma (OSCC), remains one of the most aggressive malignancies affecting the oral cavity, impacting more than 300,000 individuals worldwide each year (1). In Canada alone, approximately 5300 new cases are diagnosed annually. Despite advances in surgical techniques and multimodal therapeutic approaches, the prognosis of advanced-stage OSCC remains poor, with five-year survival rates remaining below 50% in many patient cohorts (2). Early-stage diagnosis is generally associated with improved clinical outcomes, whereas delayed detection frequently results in tumor recurrence, metastasis, and therapeutic resistance. Current standard treatments mainly rely on surgical resection followed by radiotherapy and/or platinum-based chemotherapy, including cisplatin (CDDP) and 5-fluorouracil (5-FU) (3). Although these chemotherapeutic agents are effective in reducing tumor burden, their clinical use is frequently associated with severe systemic toxicity, adverse side effects, and the progressive emergence of chemoresistance (4, 5). Consequently, there is an urgent need to identify safer and more selective therapeutic strategies capable of improving treatment efficacy while minimizing toxicity.

In recent years, naturally derived bioactive compounds have attracted increasing attention as potential anti-cancer agents. Among these compounds, flavonoids—secondary metabolites widely distributed in fruits, vegetables, and medicinal plants—have demonstrated promising anti-inflammatory, antioxidant, and anti-neoplastic properties (6). Quercetin (3,3′,4′,5,7-pentahydroxyflavone), one of the most abundant dietary flavonoids, is found in several medicinal plants, including *Ginkgo biloba* and *Sophora japonica*, which have long been used in traditional medicine for the management of inflammatory and vascular disorders (7, 8). Beyond its nutritional value, quercetin has emerged as a promising phytochemical with selective anti-cancer activity against multiple malignancies (9–11).

Several studies have demonstrated that quercetin can inhibit cancer cell proliferation through modulation of cell cycle regulators such as cyclin B1 and CDK1, ultimately promoting cell cycle arrest in different cancer models including colon, breast, ovarian, and pancreatic cancers (12–14). In addition, quercetin possesses potent antioxidant properties that contribute to the regulation of oxidative stress and the neutralization of reactive oxygen species (ROS), thereby limiting oxidative cellular damage (9, 15). Depending on the cellular context, quercetin has also been reported to modulate autophagy and apoptosis in several malignancies, including prostate cancer, lymphoblastic lymphoma, and Burkitt’s lymphoma (16–19). Furthermore, this flavonoid has been shown to suppress signaling pathways associated with angiogenesis, migration, and tumor survival while reducing the expression of epidermal growth factor (EGF)-related pathways (20).

Accumulating evidence also supports the therapeutic relevance of quercetin in oral cancer. A recent systematic review by Saadh et al. highlighted the ability of quercetin to regulate oxidative stress, apoptosis, and metastatic processes in preclinical oral cancer models (21). Additional studies demonstrated that quercetin suppresses epithelial–mesenchymal transition and inhibits the proliferation and migration of oral epidermoid carcinoma cells (22), while other investigations reported that quercetin targets the HOXA10 signaling axis to reduce oral cancer cell growth (23). In tongue squamous carcinoma models, quercetin was shown to induce apoptosis through activation of caspase-dependent pathways involving caspases 3, 8, and 9 (24). Interestingly, the effects of quercetin on oxidative stress and DNA repair mechanisms appear to be context dependent. In some cancer models, quercetin inhibits DNA repair pathways and promotes apoptosis (25), whereas in others it exhibits protective antioxidant activity against oxidative DNA damage (26, 27). Previous studies in breast, lung, and prostate cancer cells further reported increased expression of DNA damage markers such as H2AX following quercetin exposure (28). Since H2AX phosphorylation is strongly associated with DNA double-strand breaks and DNA repair signaling, these findings suggest that quercetin may interfere with genomic stability in cancer cells (29). Moreover, quercetin exerts anti-inflammatory effects through inhibition of several signaling pathways, including NF-κB and cytokine-mediated pathways, while also modulating immune responses involving T lymphocytes, dendritic cells, and macrophages (30).

Although the anti-proliferative effects of quercetin have been investigated in several oral cancer models, its selective activity across distinct OSCC subtypes and its impact on interconnected pathways such as apoptosis, autophagy, oxidative stress, DNA damage, and metastatic signaling remain insufficiently characterized. In addition, few studies have evaluated the potential of quercetin to enhance the efficacy of conventional chemotherapy agents in gingival squamous cell carcinoma. Therefore, the present study aimed to: **(i)** evaluate the selective anti-proliferative activity of quercetin against gingival and tongue squamous carcinoma cells compared with non-malignant oral epithelial cells; **(ii**) investigate its effects on apoptosis, autophagy, oxidative stress, DNA damage, and migratory potential; and **(iii)** assess its potential synergistic activity in combination with cisplatin and 5-fluorouracil.

## 2 Materials and methods

### 2.1 Cell culture

Oral squamous gingival carcinoma (Ca9-22), tongue carcinoma (SCC-9 and CAL-27), and non-oncogenic oral epithelial (GMSM-K) cell lines were exposed to increasing concentrations of quercetin (Sigma Aldrich, Oakville, Ontario, Canada) ranging from 10 to 100 µM. Ca9–22 cells were obtained from the RIKEN BioResource Research Center (Tsukuba, Japan), whereas GMSM-K cells were kindly provided by Dr. Daniel Grenier (Laval University, Quebec, Canada). SCC-9 and CAL-27 tongue carcinoma cell lines were purchased from Cedarlane Corporation (Burlington, Ontario, Canada), the local distributor of ATCC products.

Ca9–22 cells were cultured in RPMI-1640 medium (Thermo Fisher Scientific, Burlington, ON, Canada) supplemented with 5% fetal bovine serum (FBS), penicillin-streptomycin, and fungizone (Sigma Aldrich). GMSM-K, CAL-27, and SCC-9 cells were maintained in DMEM supplemented with 10% FBS. All cell lines were cultured at 37 °C in a humidified atmosphere containing 5% CO_2_. Upon reaching 80–100% confluence, cells were washed twice with phosphate-buffered saline (PBS), detached using 0.05% or 0.25% trypsin (Thermo Fisher Scientific), and counted using a hemocytometer.

### 2.2 MTT viability and proliferation assay

The effects of quercetin, either alone or in combination with cisplatin (CDDP) or 5-fluorouracil (5-FU), on cell viability and proliferation were evaluated using the MTT assay according to previously validated protocols (31–35). Briefly, 3 × 10^5^ cells were seeded into 6-well plates and exposed to increasing concentrations of quercetin (10–100 µM) for 24 h. Cells were subsequently incubated with MTT solution (5 mg/mL; Sigma Aldrich) diluted 1:10 in culture medium for 3 h at 37 °C in the dark. Metabolically active cells converted MTT into insoluble purple formazan crystals, which were dissolved using 0.04 M isopropanol-HCl solution. Optical density (OD) was measured at 550 nm using an iMark microplate spectrophotometer (Bio-Rad, Mississauga, ON, Canada).

Cell viability was calculated using the following formula:


$$\text{Cell viability }(\%)=\frac{\left[{\text{OD}}_{550}(\text{treated cells})-{\text{OD}}_{blank}\right]}{\left[{\text{OD}}_{550}(\text{control})-{\text{OD}}_{blank}\right]}\times 100$$

Experiments were independently repeated between 3 and 14 times. Combination index (CI) values were calculated using CompuSyn software. CI < 1 indicated synergistic effects, CI = 1 additive effects, and CI > 1 antagonistic effect.

### 2.3 LDH cytotoxicity assay

Cell cytotoxicity induced by quercetin was assessed using the LDH detection kit (Roche, Mississauga, ON, Canada) according to previously described protocols (36–38). Briefly, GMSM-K, Ca9-22, CAL-27, and SCC-9 cells were exposed to increasing concentrations of quercetin for 24 h. Culture supernatants were collected, and 50 µL from each condition was transferred in triplicate into a 96-well plate. Equal volumes of reconstituted substrate mixture containing dye solution and catalyst were then added.

Following 30 min incubation at room temperature in the dark, LDH release was quantified by measuring absorbance at 490 nm using an iMark microplate reader (Bio-Rad). Triton X-100 (0.2%) was used as a positive control corresponding to 100% cytotoxicity. Experiments were repeated between 3 and 9 times.

### 2.4 Clonogenic assay

Colony formation assays were performed according to previously established methods (31, 33, 34, 37, 39–41). Briefly, GMSM-K, SCC-9, CAL-27, and Ca9–22 cells were seeded into 6-well plates at a density of 2 × 10³ cells/well and treated with quercetin (0, 10, 50, and 100 µM) for two weeks. Colonies were washed twice with PBS, fixed with cold methanol for 10 min, and stained with 0.5% crystal violet for 10 min at room temperature.

Following washing and drying, colony images were acquired using a Nikon ECLIPSE TS100 microscope. Crystal violet staining was dissolved in pure acetic acid, and absorbance was measured at 570 nm using an iMark reader (Bio-Rad). Experiments were independently repeated at least four times.

### 2.5 Annexin V/Propidium iodide assay for apoptosis detection

Apoptosis was analyzed by flow cytometry using the Annexin V (Anx V)/Propidium iodide (PI) apoptosis detection kit (BD Bioscience, Mississauga, ON, Canada), as previously described (31, 33–35, 37–39, 41, 42). Ca9–22 cells were seeded in Petri dishes at 1 × 10^6^ cells/dish and allowed to adhere for 24 h prior to treatment with quercetin (0, 10, 50, and 100 µM) for an additional 24 h.

Cells were harvested, washed, and stained with 5 µL Annexin V/APC and 10 µL PI/PE according to the manufacturer’s instructions. Following 15 min incubation in the dark, 400 µL Annexin V binding buffer was added, and samples were analyzed using flow cytometry to determine the proportions of viable, apoptotic, and necrotic cells. Experiments were repeated four times.

### 2.6 Autophagy assay

Autophagy was evaluated using the Autophagy Detection Kit Red (ImmunoChemistry Technologies, Burlington, ON, Canada). Following exposure to quercetin (0, 10, 50, and 100 µM) for 24 h, Ca9–22 cells were detached using 0.05% trypsin and incubated with 10 µL red autophagy detection dye for 1 h in the dark. Cells were subsequently washed three times with PBS and resuspended in 500 µL autophagy buffer prior to flow cytometry analysis using the BD Accuri™ C6 system. Experiments were independently repeated four times.

### 2.7 Oxidative stress assessment by flow cytometry

Oxidative stress parameters were evaluated following exposure of Ca9–22 cells to increasing concentrations of quercetin (0, 10, 50, and 100 µM) for 24 h. Total intracellular ROS levels were measured using the ROS detection kit (ImmunoChemistry Technologies, Burlington, ON, Canada) (33, 37, 39, 43, 44), whereas mitochondrial superoxide production was assessed using MitoSOX Red (Molecular Probes, Invitrogen). Intracellular glutathione (GSH) levels were evaluated using the ThioBright Green assay kit (ImmunoChemistry Technologies, Bloomington, MN, USA) (34).

Cells were incubated with fluorescent probes for 1 h at 37 °C in the dark before fluorescence intensities were analyzed using the BD Accuri™ C6 flow cytometer. The percentage of positive cells was subsequently determined. All experiments were independently repeated four times.

### 2.8 DNA damage assessment by flow cytometry

DNA damage was evaluated through detection of phosphorylated H2AX (γH2AX) in quercetin-treated Ca9–22 cells. Following treatment, cells were fixed with 75% ethanol for 15 min and permeabilized using a solution containing 1% BSA, 0.2% Triton X-100, and PBS. Cells were then incubated overnight at 4 °C with monoclonal anti-phospho-H2AX (Ser139) antibody (Santa Cruz Biotechnology, Santa Cruz, CA, USA) diluted 1:100.

After washing, cells were incubated with Alexa Fluor 488-conjugated secondary antibody (1:100) for 1 h. Fluorescence intensity was analyzed using the BD Accuri™ C6 flow cytometer. Experiments were independently repeated four times.

### 2.9 Scratch migration assay

Cell migration was evaluated using a wound-healing assay. Ca9–22 cells were seeded into 6-well plates at 4 × 10^5^ cells/well and cultured until confluence. Following treatment with quercetin (0, 10, 50, and 100 µM), linear scratches were generated using sterile 200 µL pipette tips. Images were captured at 0, 6, and 24 h using an inverted optical microscope. At the endpoint, cells were stained with crystal violet.

Scratch closure was quantified using Lumenera Infinity Analyze software (version 6.5.5). Experiments were independently repeated four times.

### 2.10 Real-time PCR and RT² profiler PCR array

Total RNA extraction was performed using the RNeasy Mini Kit (Qiagen, Toronto, ON, Canada) according to the manufacturer’s instructions. RNA concentration and purity were determined using a NanoDrop 8000 spectrophotometer (Thermo Fisher Scientific). Reverse transcription was performed using the iScript™ Reverse Transcription Supermix for RT-qPCR (Bio-Rad) (37, 39).

Real-time PCR amplification was conducted using IQ™ SYBR Green Supermix (Bio-Rad) and the primer sequences listed in **Table 1**. Gene expression levels were normalized against GAPDH and/or β-actin using the 2^−^ΔΔCT method. Three technical replicates and four biological replicates were analyzed.

**Table 1:** Primer sequences used for qRT-PCR to evaluate the expression of pro-inflammatory cytokine IL-1β and MMPs genes after stimulation of Ca9–22 cells with 10 µM of Quercetin.

*Genes*	Primer sequences
IL-1β	Forward: 5′-CTGTCCTGCGTGTTGAAAGA-3′ Reverse: 5′-TTGGGTAATTTTTGGGATCTACA-3′
pro-MMP1	Forward: 5′-GATCATCGGGACAACTCTCCT-3′ Reverse: 5′-TCCGGGTAGAAGGGATTTGTG-3′
pro-MMP2	Forward: 5′-TTGGCAGCCTTCCTGATT -3′ Reverse: 5′-AACTTCTCCACAACCCTCTG-3′
pro-MMP3	Forward: 5′-CACTGTCCACCCTCAGAGC-3′ Reverse: 5′-GCCACTTGTCGGCGATAAGG-3′
GAPDH	Forward: 5’GGTATCGTCGAAGGACTCATGAC-3’ Reverse: 5’-ATGCCATGAGCTTCCCGTTCAGC-3’′

The RT² Profiler PCR Array Human Apoptosis Panel (Qiagen, PAHS-012ZD-6) was used to screen 84 apoptosis-related genes potentially modulated by quercetin. Transcriptomic data were analyzed using Bio-Rad CFX Manager software (version 3.1) and normalized to either GAPDH and/or β-actin. Relative gene expression data analysis was performed using the 2-ΔΔCT method. The experiment was repeated four times.

### 2.11 Gene interaction and survival analysis

Gene interaction networks were analyzed using the GeneMANIA platform (https://genemania.org/). Functional enrichment analysis was prioritized according to false discovery rate (FDR) values and coverage scores to identify biologically relevant pathways.

The prognostic significance of genes modulated by quercetin was evaluated using the UALCAN web portal based on The Cancer Genome Atlas (TCGA) head and neck squamous cell carcinoma datasets.

### 2.12 Molecular docking and molecular dynamics simulations

The crystal structures of CASP9 (PDB: 1NW9), H2AX (PDB: 6ZWK), and BIRC3 (PDB: 2UVL) were retrieved from the RCSB Protein Data Bank. Protein preparation, active site prediction, receptor grid generation, molecular docking, and molecular dynamics simulations were performed using the Schrödinger Maestro suite (Schrödinger Release 2025-2, New York, NY, USA) according to standard computational protocols.

Quercetin-protein interactions were evaluated using Glide Standard Precision (SP) flexible docking. Binding poses were selected based on docking score, interaction profile, and structural stability during molecular dynamics simulations.

### 2.13 Statistical analysis

All experiments were performed at least three independent times, and results are expressed as mean ± SEM or SD, as indicated in figure legends. Statistical analyses were performed using one-way ANOVA followed by appropriate *post hoc* tests. A p-value < 0.05 was considered statistically significant.

## 3 Results

### 3.1 Quercetin selectively inhibits oral cancer cell proliferation with greater efficacy against gingival carcinoma cells

To evaluate the anti-proliferative effects of quercetin in oral cancer models, gingival squamous carcinoma (Ca9-22), tongue carcinoma (SCC-9 and CAL-27), and non-malignant oral epithelial (GMSM-K) cells were exposed to increasing concentrations of quercetin (0–100 µM). As shown in **Figure 1A**, quercetin induced a pronounced dose-dependent reduction in Ca9–22 cell viability, whereas SCC-9, CAL-27, and GMSM-K cells displayed significantly lower sensitivity to treatment. At 50 µM, quercetin reduced Ca9–22 viability by approximately 50%, while viability reduction remained below 20% in SCC-9, CAL-27, and GMSM-K cells. At 100 µM, Ca9–22 viability decreased to less than 20%, confirming the strong susceptibility of gingival carcinoma cells to quercetin treatment.

**Figure 1:**
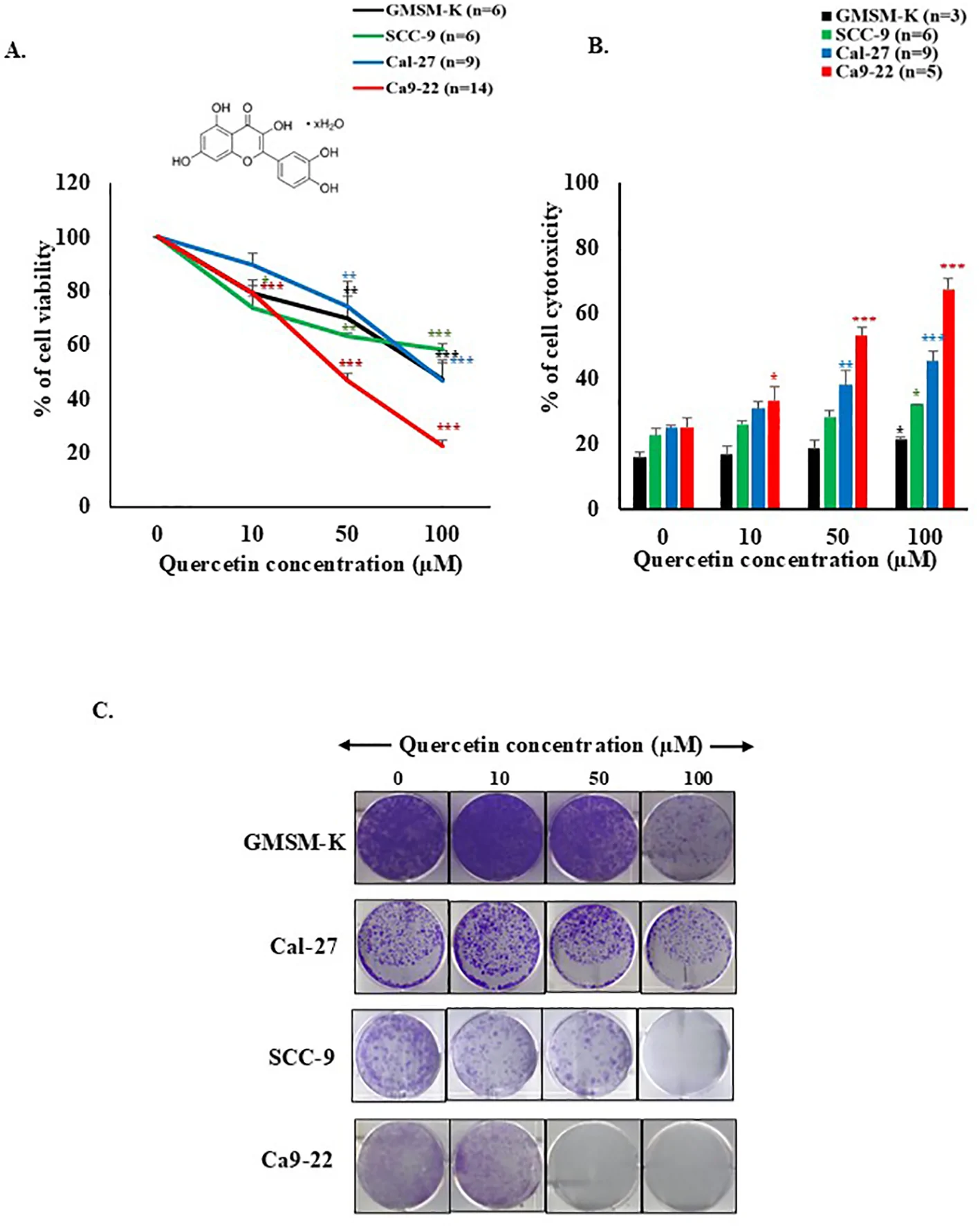
Quercetin inhibits oral cancer cells proliferation and colony formation. GMSM-k, CAL27, SCC9 and Ca9–22 were exposed to growing concentrations (0, 10, 50 and 100 µM) of Quercetin. After 24h, **(A)** cell viability was evaluated by the means of the MTT assay, and **(B)** cytotoxicity was measured using the LDH assay. **(C)** Colony formation potential was assessed using crystal violet staining after two weeks exposure to the same Quercetin concentrations. Data is represented as means of percentage ± SD. *p <0.05, **p <0.01, and *** p <0.001 were considered as statistically significant.

Similarly, LDH cytotoxicity assays demonstrated a significant dose-dependent increase in cytotoxicity in Ca9–22 cells following quercetin exposure (**Figure 1B**). Notably, increased cytotoxicity was already detectable at 10 µM in Ca9–22 cells, whereas tongue carcinoma and non-malignant epithelial cells remained considerably less affected. Based on CompuSyn analysis, the IC50 value of quercetin in Ca9–22 cells ranged between 24.66 and 28.24 µM. Importantly, quercetin exerted limited cytotoxicity toward non-malignant oral epithelial cells, suggesting a potentially favorable therapeutic index.

Long-term clonogenic assays further confirmed the selective anti-proliferative activity of quercetin. Exposure to 50 µM quercetin for two weeks almost completely abolished colony formation in Ca9–22 cells, while SCC-9, CAL-27, and GMSM-K cells retained substantial clonogenic capacity (**Figure 1C**). Collectively, these findings demonstrate that quercetin selectively suppresses gingival carcinoma cell proliferation and survival.

### 3.2 Quercetin induces apoptosis in gingival carcinoma cells

Given the marked sensitivity of Ca9–22 cells to quercetin, we next investigated whether apoptosis contributed to its anti-cancer effects. Flow cytometry analysis using Annexin V/PI staining demonstrated a significant increase in apoptotic and dead cell populations following quercetin treatment (**Figure 2**). Exposure to 100 µM quercetin increased the proportion of dead cells from 3.77% in untreated controls to 16.1% (**Figures 2A, B**).

**Figure 2:**
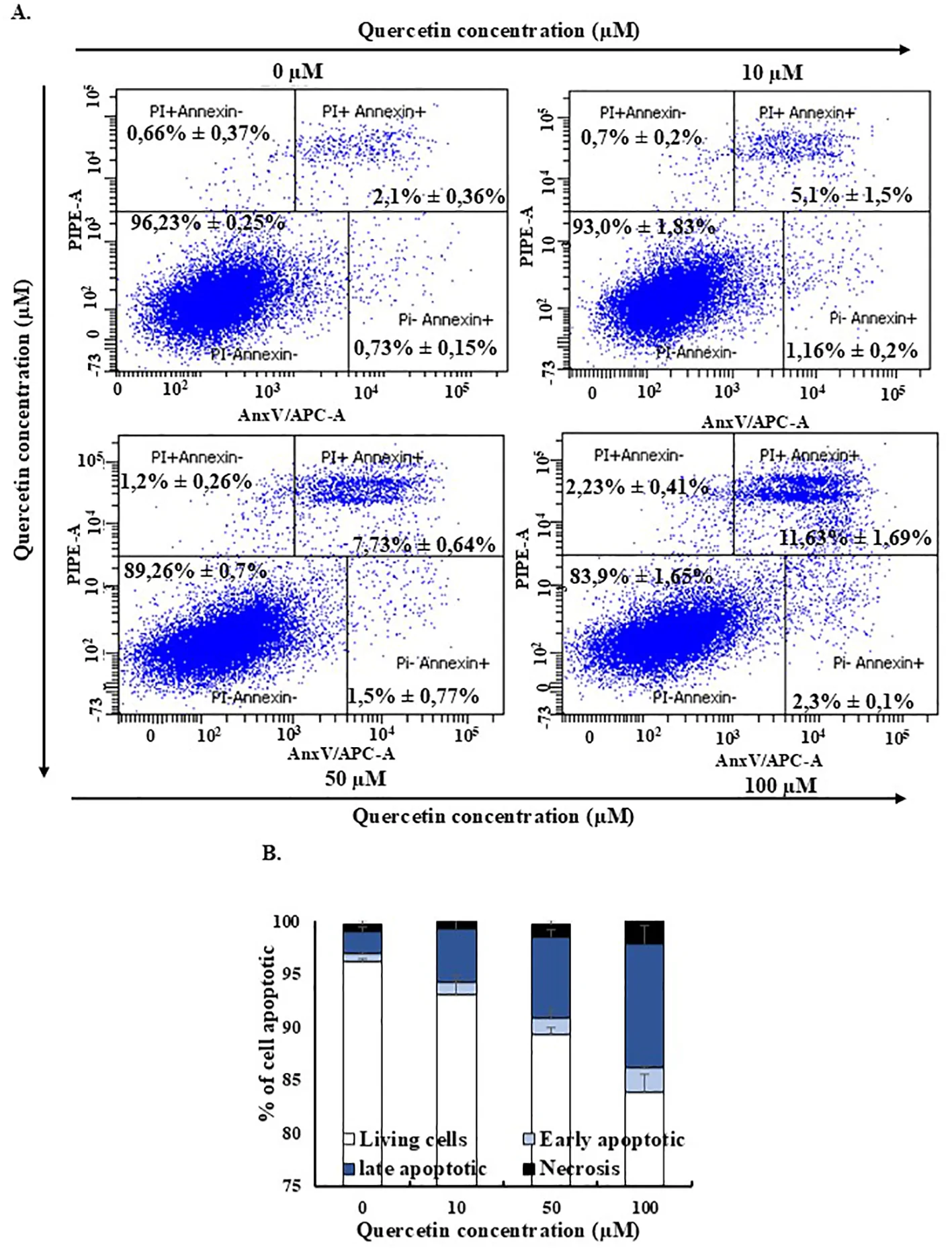
Quercetin activates Ca9–22 cell apoptosis. Quercetin effect on cell apoptosis was evaluated by flow cytometry using the Annexin V/Propidium iodide assay. **(A)** Percentage of dead cells populations (PI-/Anx+ early apoptotic, PI+/Anx+ late apoptotic, and PI+/Anx- necrotic) detected in controls (0.1% DMSO) as well as in cells treated with Quercetin at 10, 50, and 100 µM was recorded after 24 h treatment period. **(B)** Representative data corresponds to 4 independent experiments. Data is presented as means ± SD.

To further characterize the molecular mechanisms underlying quercetin-induced apoptosis, a transcriptomic analysis screening 84 apoptosis-related genes was performed using an RT² Profiler PCR array. Treatment with 50 µM quercetin induced significant modulation of several apoptosis-associated genes in Ca9–22 cells (**Figure 3A**). Genes exhibiting more than two-fold changes included APAF1, BIRC2, BIRC3, BNIP3L, CASP2, CASP4, CASP9, FAS, TRADD, and TRAF2, whereas BIRC5 expression was significantly downregulated. Among these markers, BCL2A1 exhibited the strongest upregulation (8.92-fold) following quercetin exposure (**Table 1**).

**Figure 3:**
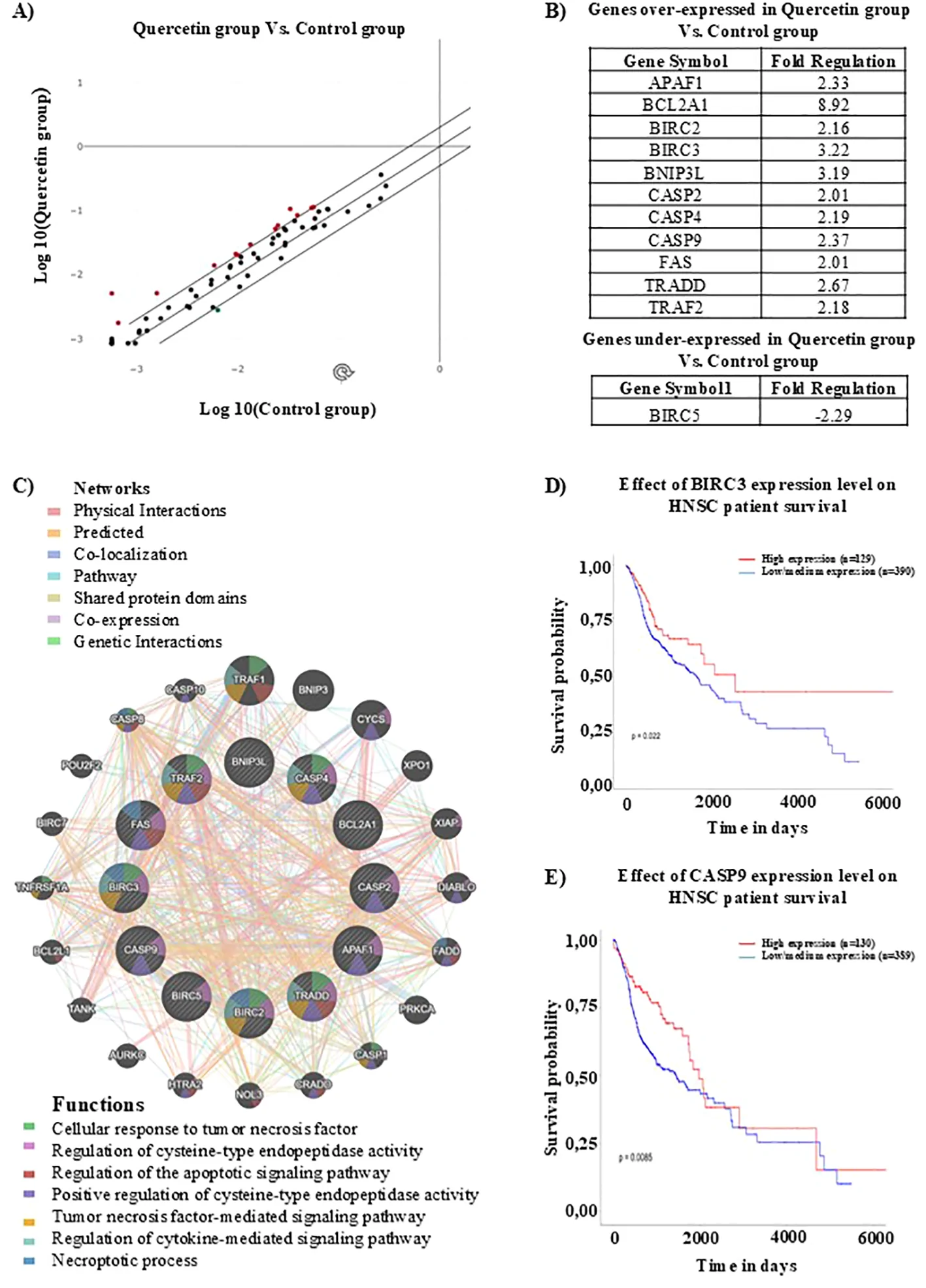
Genetic signature associated with Quercetin apoptotic effects. Ca9–22 cells were treated with 50 µM of Quercetin for 24h. Following RNA extraction and reverse transcription, a transcriptomic analysis screening for 84 apoptotic genes was performed. **(A)** Scatter plot analysis, as well as **(B)** List of modulated genes are presented. Data corresponds to one representative study performed by QPCR array. **(C)** Network analysis and identification of gene interaction networks realized using the GeneMania tool (https://genemania.org/). Comprehensive analysis of the prognostic impact of **(D)** BIRC3 and **(E)** CASP9 genes upregulation.

These findings suggest simultaneous activation of intrinsic and extrinsic apoptotic pathways in response to quercetin treatment. Gene interaction network analysis further revealed that the modulated apoptotic genes interact with multiple regulators involved in apoptosis signaling, TNF-mediated responses, necroptosis, and caspase activation pathways (**Figure 3B**). Functional enrichment analysis highlighted pathways associated with cytokine-mediated apoptotic signaling and regulation of cysteine-type endopeptidase activity.

Interestingly, survival analysis using TCGA head and neck squamous cell carcinoma datasets identified BIRC3 and CASP9 as clinically relevant prognostic markers. Elevated expression of both genes significantly correlated with improved overall patient survival (**Figures 3C, D**), supporting the translational relevance of quercetin-mediated apoptotic signaling in oral cancer.

### 3.3 Quercetin suppresses autophagy in gingival carcinoma cells

Because autophagy is increasingly recognized as a key regulator of tumor progression and therapeutic resistance, we evaluated the effects of quercetin on autophagic activity in Ca9–22 cells. Flow cytometry analysis using an autophagy detection dye demonstrated that quercetin significantly reduced autophagic activity in a dose-dependent manner (**Figures 4A, B**).

**Figure 4:**
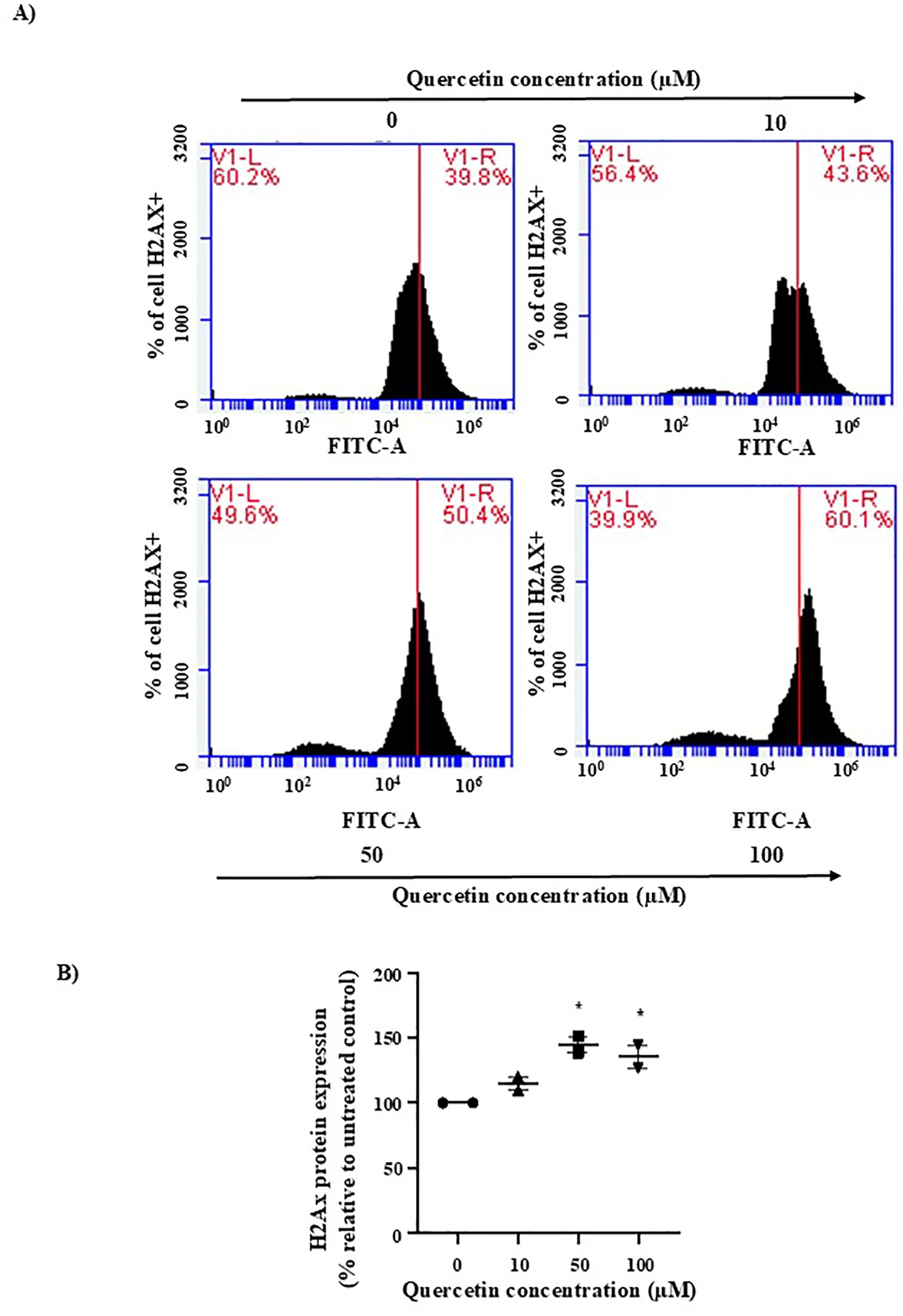
Quercetin reduces the autophagic potential of Ca9–22 cells. Autophagy was assessed by flow cytometry using a red autophagy detection dye. **(A)** Representative flow cytometry plots, and **(B)** Quantification of autophagic cells percentage, compiled from four independent experiments. Data are presented as mean ± SD. **p <0.01, and *** p <0.001 were considered statistically significant.

More specifically, treatment with 50 and 100 µM quercetin decreased the percentage of autophagic cells from 96.17% ± 1.81 in untreated controls to 83.42% ± 1.55 and 79.3% ± 1.75, respectively. These findings suggest that quercetin suppresses autophagy in gingival carcinoma cells, potentially contributing to its anti-cancer activity. Since sustained autophagy has been associated with tumor survival and therapy resistance in advanced cancers, autophagy inhibition may represent an important mechanism underlying quercetin-mediated cytotoxicity.

### 3.4 Quercetin reduces oxidative stress in gingival carcinoma cells

The impact of quercetin on oxidative stress was subsequently investigated in Ca9–22 cells. Total intracellular ROS levels were significantly reduced following quercetin exposure in a dose-dependent manner (**Figures 5A, B**). ROS-positive cells decreased from 66.4% in untreated controls to 9.7% and 7.1% after treatment with 50 and 100 µM quercetin, respectively.

**Figure 5:**
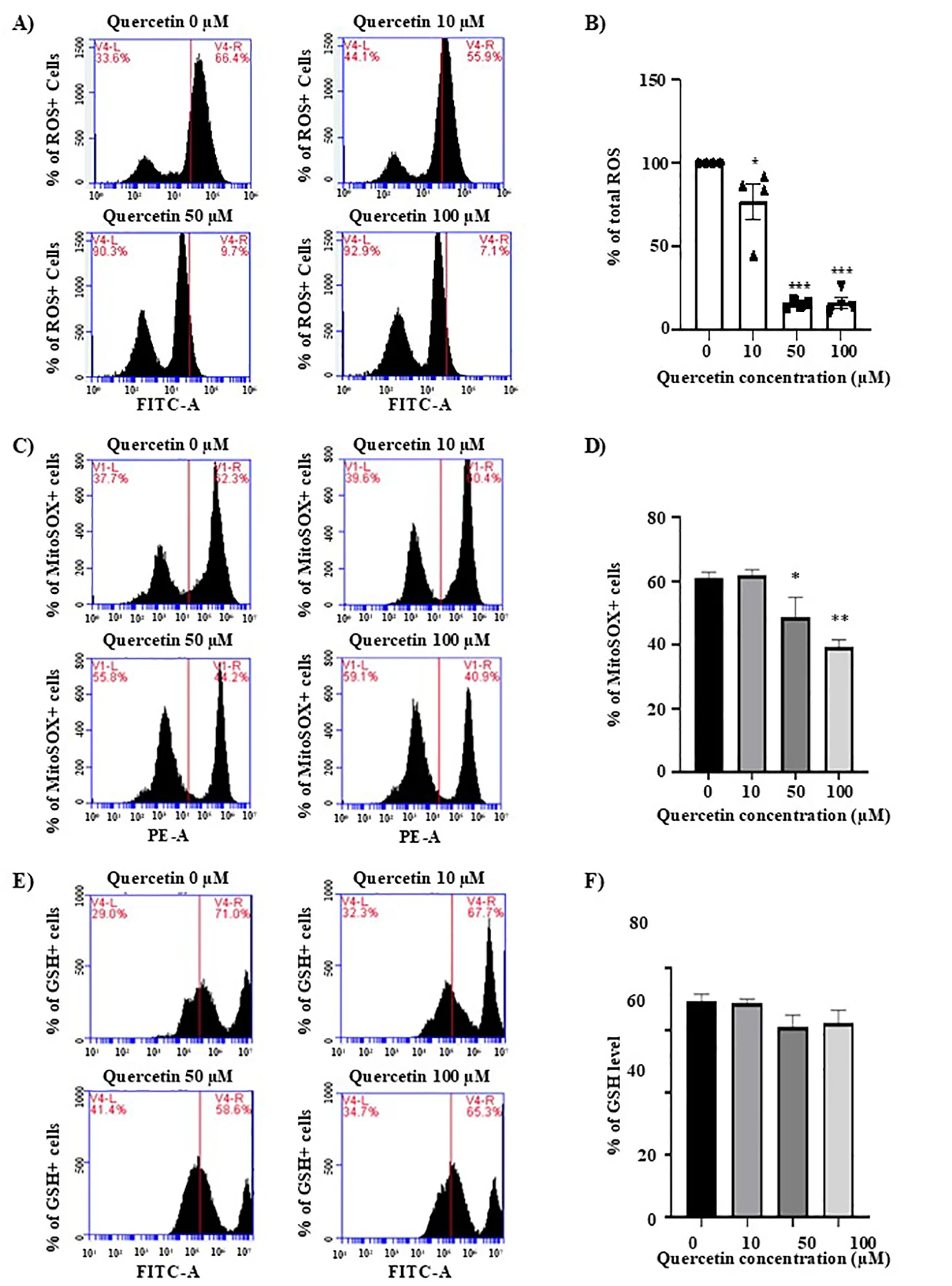
Quercetin inhibits oxidative stress in gingival carcinoma cells. **(A, B)** Total intracellular ROS levels, **(C, D)** mitochondrial ROS (MitoSOX), and **(E, F)** intracellular antioxidant GSH levels in Ca9–22 cells after 24h exposure to increasing Quercetin concentrations (0, 10, 50, and 100 µM). Control cells were treated with 0.1% DMSO. The percentage of positive cells was determined by flow cytometry. Data are expressed as mean ± SD from four independent experiments. *p <0.05, **p <0.01, and *** p <0.001 were considered as statistically significant.

Similarly, mitochondrial oxidative stress assessed using MitoSOX staining was significantly attenuated following quercetin treatment (**Figures 5C, D**). MitoSOX-positive cells decreased from 61.05% ± 1.25 in controls to 48.6% ± 4.45 and 39.3% ± 1.52 following exposure to 50 and 100 µM quercetin, respectively.

In contrast, intracellular glutathione (GSH) levels were not significantly altered by quercetin treatment (**Figures 5E, F**). These observations indicate that quercetin exerts substantial antioxidant effects in gingival carcinoma cells. The anti-cancer activity of quercetin may therefore involve complex redox modulation affecting mitochondrial homeostasis, apoptosis signaling, and DNA integrity rather than simple ROS overproduction.

### 3.5 Quercetin promotes DNA damage in gingival carcinoma cells

To determine whether quercetin affects genomic stability in gingival carcinoma cells, DNA damage was evaluated through quantification of phosphorylated H2AX (γH2AX), a well-established marker of DNA double-strand breaks. Flow cytometry analysis revealed a significant dose-dependent increase in γH2AX levels following quercetin exposure (**Figures 6A, B**).

**Figure 6:**
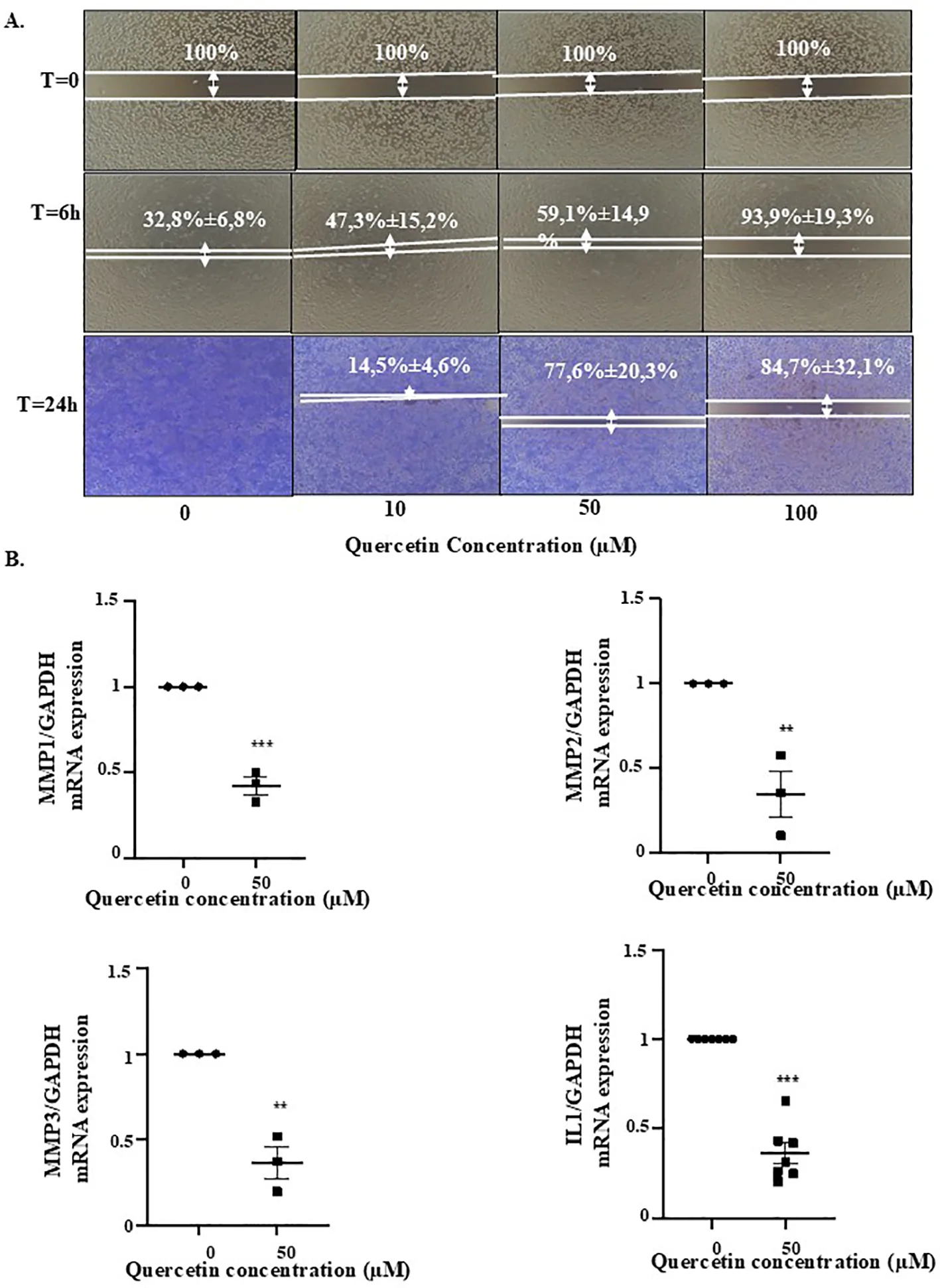
Quercetin induces DNA damage in Ca9–22 cells. **(A)** Representative flow cytometry plots, and **(B)** Quantification of phosphorylated H2AX levels following exposure to increasing concentrations of Quercetin (0, 10, 50, and 100 µM) for 24h. Control cells were treated with 0.1% DMSO. The percentage of positive cells was determined by flow cytometry. Data are expressed as mean ± SD from four independent experiments. *p <0.05 was considered as statistically significant.

The percentage of γH2AX-positive cells increased from 39.8% in untreated controls to 50.4% and 60.1% following treatment with 50 and 100 µM quercetin, respectively. Persistent H2AX phosphorylation suggests accumulation of DNA double-strand breaks and potentially ineffective DNA repair processes in quercetin-treated cells. These findings indicate that quercetin contributes to genomic stress and DNA damage signaling in gingival carcinoma cells.

### 3.6 Quercetin inhibits the migratory and metastatic potential of gingival carcinoma cells

The anti-metastatic potential of quercetin was investigated using wound-healing migration assays and gene expression analysis of metastasis-associated markers. Untreated Ca9–22 cells rapidly migrated and almost completely closed the wound area within 24 h (**Figure 7A**). In contrast, cells treated with 100 µM quercetin exhibited only 15.3% wound closure after 24 h, indicating strong inhibition of migratory capacity. Significant differences in migration were already detectable after 6 h of treatment.

**Figure 7:**
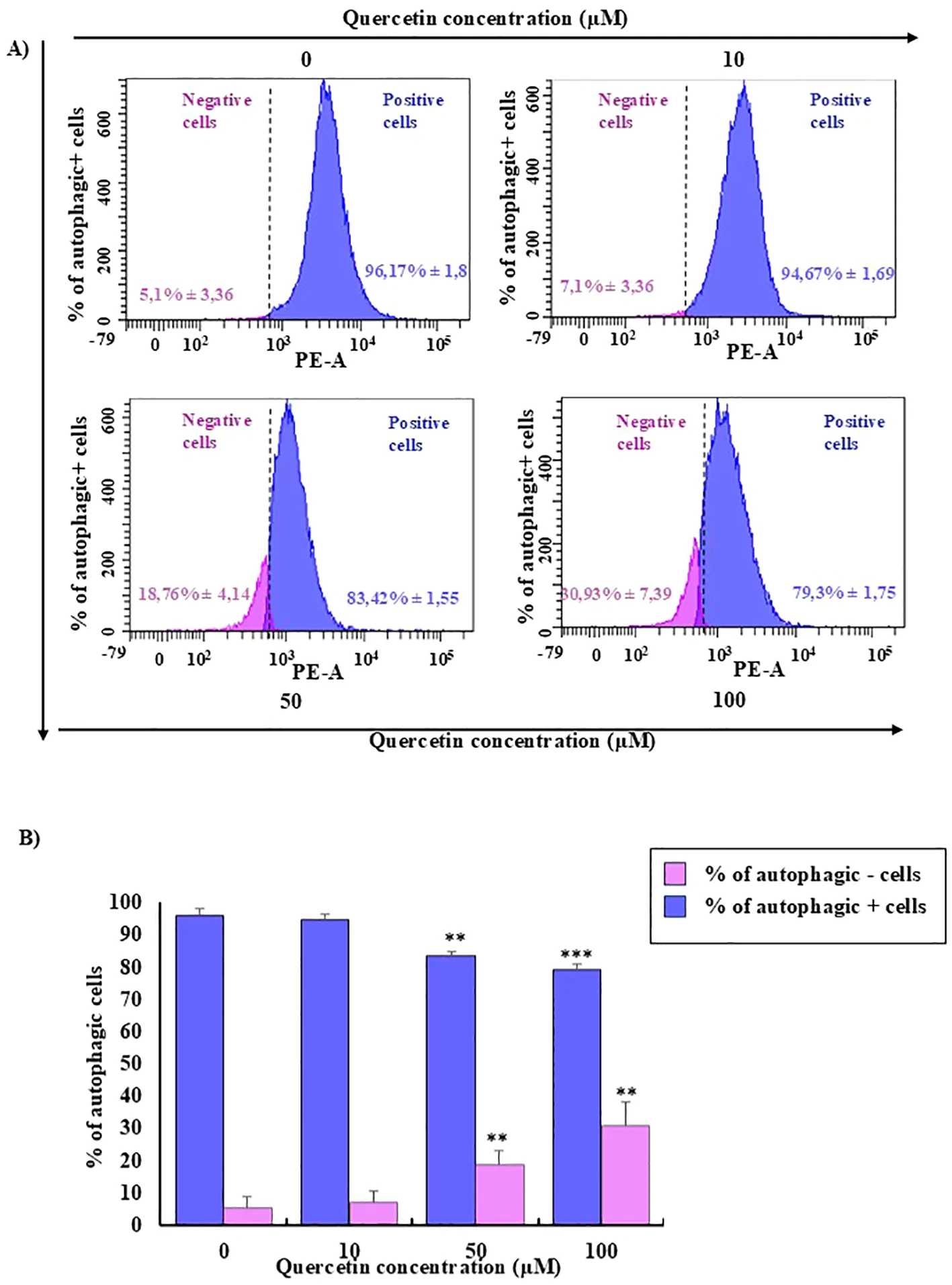
Quercetin reduces the migration potential of gingival carcinoma cells. **(A)** Evaluation of scratch diameter after 6 and 24h exposure to Quercetin. **(B)** PCR analysis of MMP1, MMP2, MMP3 and IL-1β expression levels following treatment with 50 µM of Quercetin. Data are expressed as mean ± SD from three independent experiments. **p <0.01, and *** p <0.001 were considered statistically significant.

To further investigate the molecular basis of migration inhibition, expression levels of metastasis-related genes were analyzed by RT-qPCR. Quercetin significantly downregulated the expression of MMP1, MMP2, MMP3, and the pro-inflammatory cytokine IL-1β (**Figure 7B**). Since matrix metalloproteinases are critical mediators of extracellular matrix remodeling and metastatic dissemination, these findings suggest that quercetin suppresses metastatic signaling pathways in gingival carcinoma cells.

### 3.7 Quercetin enhances the anti-proliferative effects of cisplatin and 5-fluorouracil

The potential of quercetin to enhance conventional chemotherapy efficacy was investigated by combining quercetin with cisplatin (CDDP) or 5-fluorouracil (5-FU). MTT assays demonstrated that several quercetin-containing combinations produced stronger anti-proliferative effects than chemotherapy alone (**Figures 8A, C**).

**Figure 8:**
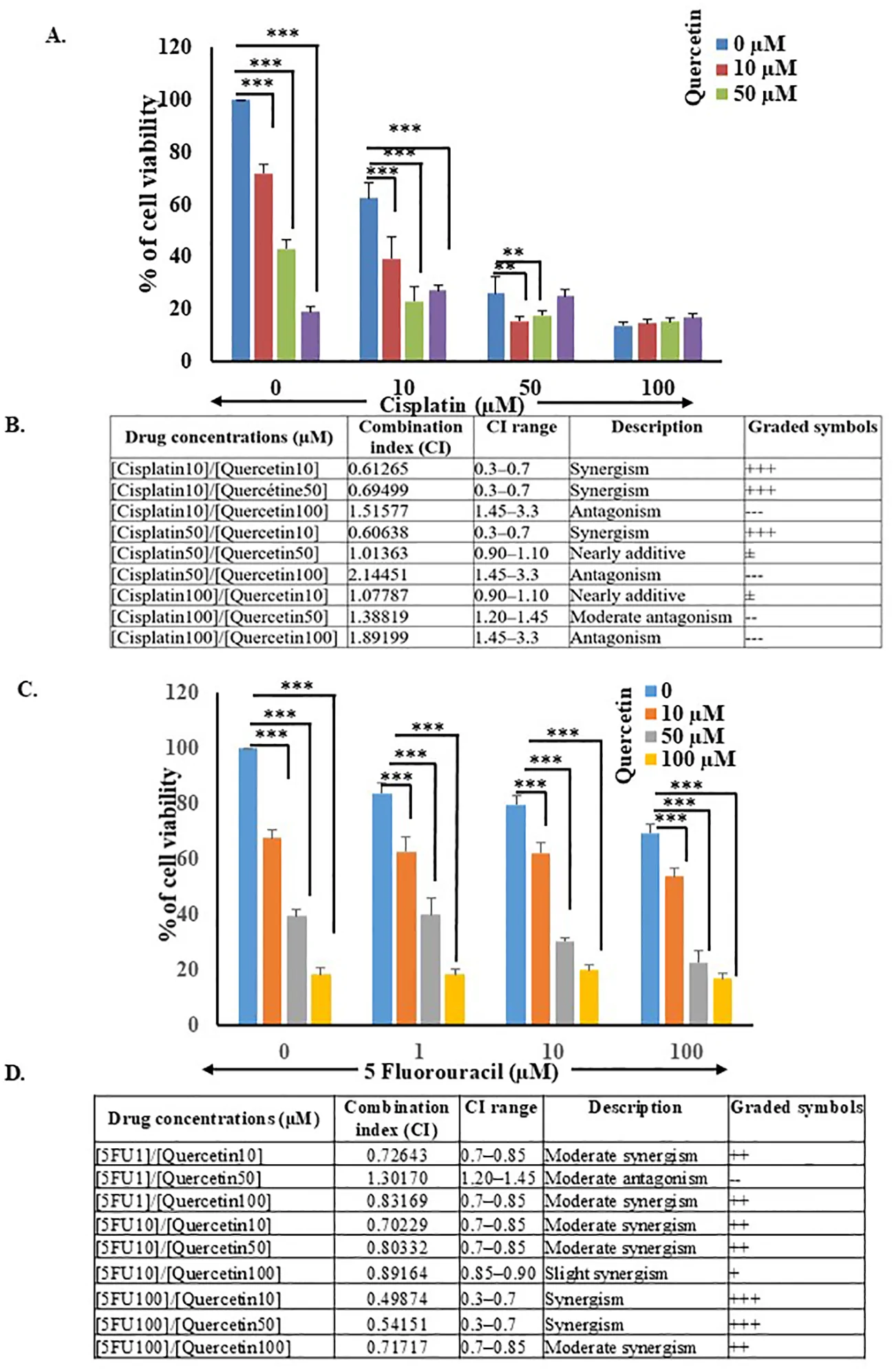
Quercetin is a potential adjuvant candidate for gingival carcinoma management. Evaluation of Ca9–22 cell viability after exposure to Quercetin whether alone or in combination with different concentrations of **(A)** CDDP and **(C)** 5-FU. Data are expressed as mean ± SD from 5 independent experiments. **p <0.01, and *** p <0.001 were considered statistically significant. Based on MTT data, **(B, D)** analysis of potential synergistic, additive, and antagonistic effects was performed using Compusyn software. CI values indicate a synergistic effect when < 1, antagonistic effect when > 1 and an additive effect when equal to 1.

Combination index (CI) analysis using CompuSyn software revealed synergistic or additive interactions between quercetin and both chemotherapeutic agents at specific concentrations (**Figures 8B, D**; **Tables 2**, **3**). However, antagonistic interactions were also observed at certain doses, highlighting the importance of treatment optimization.

**Table 2:** Effect of the combination of CDDP chemotherapy drugs with Quercetin.

Drug concentrations (μM)	Combination index (CI)	CI range	Description	Graded symbols
[Cisplatin10]/[Quercetin10]	0.61265	0.3–0.7	Synergism	+++
[Cisplatin10]/[Quercetin50]	0.69499	0.3–0.7	Synergism	+++
[Cisplatin10]/[Quercetin100]	1.51577	1.45–3.3	Antagonism	‐‐‐
[Cisplatin50]/[Quercetin10]	0.60638	0.3–0.7	Synergism	+++
[Cisplatin50]/[Quercetin50]	1.01363	0.90–1.10	Nearly additive	±
[Cisplatin50]/[Quercetin100]	2.14451	1.45–3.3	Antagonism	‐‐‐
[Cisplatin100]/[Quercetin10]	1.07787	0.90–1.10	Nearly additive	±
[Cisplatin100]/[Quercetin50]	1.38819	1.20–1.45	Moderate antagonism	‐‐
[Cisplatin100]/[Quercetin100]	1.89199	1.45–3.3	Antagonism	‐‐‐

**Table 3:** Effect of the combination of 5FU with Quercetin on Ca9-22 cells.

Drug concentrations (μM)	Combination index (CI)	CI range	Description	Graded symbols
[5FU1]/[Quercetin10]	0.72643	0.7–0.85	Moderate synergism	++
[5FU1]/[Quercetin50]	1.30170	1.20–1.45	Moderate antagonism	‐‐
[5FU1]/[Quercetin100]	0.83169	0.7–0.85	Moderate synergism	++
[5FU10]/[Quercetin10]	0.70229	0.7–0.85	Moderate synergism	++
[5FU10]/[Quercetin50]	0.80332	0.7–0.85	Moderate synergism	++
[5FU10]/[Quercetin100]	0.89164	0.85–0.90	Slight synergism	+
[5FU100]/[Quercetin10]	0.49874	0.3–0.7	Synergism	+++
[5FU100]/[Quercetin50]	0.54151	0.3–0.7	Synergism	+++
[5FU100]/[Quercetin100]	0.71717	0.7–0.85	Moderate synergism	++

These findings indicate that quercetin may function as a chemosensitizing agent capable of potentiating cisplatin and 5-FU efficacy in gingival carcinoma cells. Such combinations may ultimately support dose-reduction strategies aimed at minimizing chemotherapy-associated toxicity.

### 3.8 Quercetin interacts with CASP9, H2AX, and BIRC3 and forms stable protein–ligand complexes

To further investigate the molecular basis of quercetin activity, molecular docking and molecular dynamics simulations were performed using CASP9, H2AX, and BIRC3 proteins. Docking analyses demonstrated that quercetin efficiently binds within SiteMap-predicted pockets of all three target proteins through multiple hydrogen bonds, hydrophobic contacts, and aromatic interactions (**Figure 9**).

**Figure 9:**
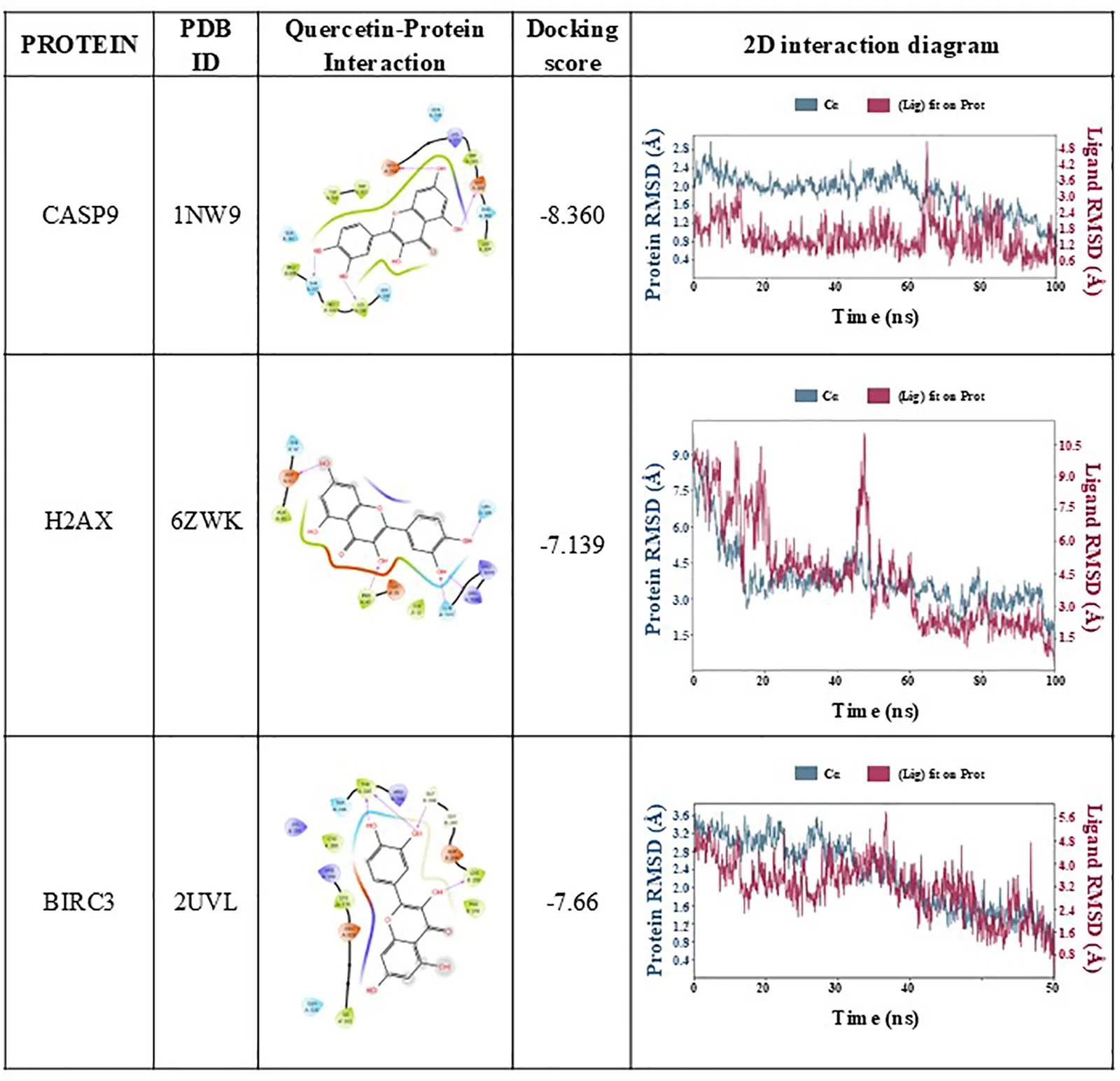
Quercetin interacts with CASP9, H2AX and BIRC3. Protein–ligand interaction profiles and dynamic stability of quercetin bound to CASP9 (PDB ID: 1NW9), H2AX (PDB ID: 6ZWK) and BIRC3 (PDB ID: 2UVL) are presented. The 2D interaction diagrams depict quercetin binding within SiteMap-predicted pockets. Hydrophobic interactions (blue), hydrogen bonds (green), and water bridges (purple) are highlighted due to their contributions to ligand stabilization. Protein–ligand complex stability was assessed over a 50 to 100 ns molecular dynamics simulation. Protein backbone RMSD is shown in blue (left y-axis), and the ligand RMSD, calculated after fitting to the protein backbone (right y-axis; maroon), reflects quercetin binding pose within the predicted pocket.

In CASP9, quercetin established stable interactions involving residues such as GLU314, SER339, THR338, THR355, and TRP340. Similarly, stable polar and hydrophobic interactions were observed within the H2AX binding pocket, notably involving ASN100, ASN102, SER62, and PHE41. In BIRC3, quercetin formed multiple hydrogen bonds with residues including TYR245, ARG244, ASP296, and GLU320, supporting stable ligand accommodation within the apoptotic regulatory domain.

Molecular dynamics simulations further confirmed the stability of quercetin–protein complexes over 50–100 ns simulation periods. RMSD trajectories demonstrated limited conformational fluctuations for both proteins and ligands, indicating stable protein–ligand interactions throughout the simulations. Collectively, these computational findings support the biological relevance of quercetin interactions with apoptosis- and DNA damage-related proteins.

## 4 Discussion

Oral squamous cell carcinoma (OSCC) remains a major therapeutic challenge due to tumor recurrence, metastatic dissemination, chemoresistance, and the severe adverse effects associated with conventional therapies. These limitations highlight the urgent need for safer and more selective therapeutic approaches capable of improving treatment efficacy while minimizing toxicity. In the present study, we investigated the anti-cancer effects of quercetin, a naturally occurring flavonoid, in different oral cancer models and demonstrated, for the first time, a marked selective activity toward gingival squamous carcinoma cells. More specifically, quercetin suppressed gingival carcinoma cell proliferation and clonogenicity while exerting limited cytotoxicity toward non-malignant oral epithelial cells. This selective anti-tumor activity was associated with coordinated modulation of apoptosis, autophagy, oxidative stress, DNA damage, migration, and chemotherapy sensitivity.

Quercetin is widely recognized for its antioxidant and anti-inflammatory properties and has increasingly attracted attention for its anti-cancer potential (4, 45). Previous investigations reported that quercetin inhibits proliferation and induces apoptosis in multiple cancer types, including oral carcinoma (4, 13). Our findings are consistent with these studies and further demonstrate that gingival carcinoma cells exhibit significantly greater sensitivity to quercetin compared with tongue carcinoma and non-malignant epithelial cells. This observation suggests the existence of subtype-specific vulnerabilities within oral squamous cell carcinoma that may influence therapeutic responsiveness to natural compounds. Importantly, the selective cytotoxicity observed in Ca9–22 cells supports the possibility of a favorable therapeutic index, an essential characteristic for future translational applications.

One of the major findings of the present study is the ability of quercetin to induce apoptosis in gingival carcinoma cells. Programmed cell death represents a central anti-cancer mechanism targeted by many conventional therapies (46, 47). Flow cytometry analysis confirmed increased apoptotic and dead cell populations following quercetin exposure, while transcriptomic profiling revealed modulation of multiple apoptosis-related genes. More specifically, quercetin upregulated several key mediators involved in intrinsic and extrinsic apoptotic pathways, including APAF1, CASP2, CASP4, CASP9, FAS, TRADD, and TRAF2. APAF1 plays a critical role in apoptosome assembly and caspase activation, and its downregulation is frequently associated with tumor progression and resistance to apoptosis (48, 49). Therefore, APAF1 upregulation may contribute to the restoration of apoptotic sensitivity in gingival carcinoma cells.

Similarly, quercetin induced the expression of BNIP3L, a mitochondrial outer membrane protein implicated in apoptosis and mitophagy regulation (50). The activation of caspase-related genes further supports the involvement of mitochondrial apoptotic signaling pathways. Interestingly, quercetin also increased the expression of FAS, a death receptor frequently downregulated in cancer cells (51), suggesting activation of extrinsic apoptotic signaling. In contrast, BIRC5 (survivin), a well-known anti-apoptotic molecule associated with poor prognosis and therapy resistance (52), was significantly downregulated following quercetin treatment. Collectively, these findings suggest that quercetin promotes apoptosis through simultaneous activation of multiple pro-apoptotic pathways while suppressing survival-associated signaling.

The transcriptomic analysis also revealed upregulation of BIRC2, BIRC3, and BCL2A1, genes often associated with anti-apoptotic responses and treatment resistance (53). Although these molecules may represent compensatory survival mechanisms activated in response to cellular stress, survival analysis using TCGA datasets demonstrated that elevated BIRC3 and CASP9 expression correlated with improved patient survival in head and neck squamous cell carcinoma. These observations suggest that the molecular effects of quercetin may possess translational relevance in oral oncology. Nevertheless, additional validation studies at the protein level would be required to further characterize the precise functional significance of these transcriptomic alterations.

Autophagy is increasingly recognized as a critical regulator of cancer progression and therapeutic resistance (54, 55). However, its role in cancer remains highly context dependent. While autophagy may suppress tumor initiation in early carcinogenesis, sustained autophagy in advanced cancers often supports metabolic adaptation, tumor survival, and resistance to therapy (56). In the present study, quercetin significantly reduced autophagic activity in gingival carcinoma cells. These findings suggest that autophagy inhibition may contribute to the anti-cancer effects of quercetin in advanced oral carcinoma. Similar observations have previously been reported with natural compounds capable of sensitizing oral cancer cells to chemotherapy through autophagy inhibition (57, 58). Interestingly, quercetin has also been reported to induce autophagy in other malignancies (19, 59–61), further emphasizing the context-dependent nature of autophagic regulation. Because the current study primarily evaluated autophagic vacuole accumulation, future investigations assessing LC3-II and p62 turnover would be necessary to fully characterize autophagic flux modulation by quercetin.

Oxidative stress plays a fundamental role in oral carcinogenesis and tumor progression. Cancer cells frequently exhibit elevated ROS production that contributes to genomic instability, metabolic adaptation, and resistance to apoptosis (62, 63). In the present study, quercetin significantly reduced both total intracellular ROS and mitochondrial oxidative stress in gingival carcinoma cells. These findings are consistent with the well-established antioxidant properties of quercetin (9, 15). Interestingly, despite the reduction in ROS levels, quercetin simultaneously promoted apoptosis and DNA damage signaling. This apparent paradox suggests that quercetin-mediated anti-cancer activity may not simply rely on ROS overproduction but rather on complex redox modulation affecting mitochondrial homeostasis, DNA integrity, and apoptotic signaling pathways. Similar dual effects have been previously reported for several phytochemicals capable of simultaneously regulating oxidative stress and inducing cancer cell death (46, 47).

DNA damage induction represents another important mechanism underlying the anti-cancer activity of quercetin. In this study, quercetin significantly increased phosphorylation of H2AX, a key marker of DNA double-strand breaks and genomic stress (29). Persistent γH2AX accumulation may indicate ineffective DNA repair and sustained genomic instability in treated gingival carcinoma cells. Previous studies also reported that quercetin induces oxidative DNA damage and apoptosis in different cancer models (64). Similar effects have been described for other natural compounds, including curcumin analogs (34). Consequently, pharmacological induction of DNA damage using naturally derived compounds may represent a promising strategy for selectively targeting oral cancer cells.

Metastatic dissemination remains one of the principal causes of mortality in oral cancer patients. Matrix metalloproteinases (MMPs) play essential roles in extracellular matrix remodeling, tumor invasion, and metastatic progression. In the present study, quercetin significantly inhibited Ca9–22 cell migration and reduced the expression of MMP1, MMP2, MMP3, and IL-1β. These findings suggest that quercetin interferes with metastatic signaling pathways and extracellular matrix remodeling. Similar inhibitory effects of quercetin on MMP expression and metastatic activity have previously been reported in prostate cancer and other malignancies (65, 66). Therefore, beyond its cytotoxic activity, quercetin may also contribute to suppression of oral cancer progression and dissemination.

One of the most clinically relevant observations of this study is the ability of quercetin to enhance the anti-proliferative effects of cisplatin and 5-fluorouracil. Combination index analyses revealed synergistic or additive interactions at several concentration combinations, supporting the potential use of quercetin as a chemosensitizing agent. Similar synergistic interactions between quercetin and cisplatin have previously been described in oral squamous cell carcinoma models through modulation of NF-κB and xIAP signaling pathways (67). The use of quercetin in combination therapy may therefore support future dose-reduction strategies aimed at limiting chemotherapy-associated toxicity while preserving anti-tumor efficacy.

The molecular docking and molecular dynamics analyses performed in this study further support the biological relevance of quercetin-mediated signaling modulation. Stable interactions between quercetin and apoptosis- or DNA damage-related proteins, including CASP9, H2AX, and BIRC3, suggest that quercetin may directly interact with multiple molecular targets involved in cancer cell survival and genomic stability. Although these computational analyses remain predictive, they provide additional mechanistic insights supporting the multi-target anti-cancer activity of quercetin.

Several limitations should nevertheless be acknowledged. First, this study was restricted to *in vitro* models and requires validation *in vivo* using preclinical oral cancer models. Second, although transcriptomic analyses identified multiple molecular pathways modulated by quercetin, protein-level validation was not comprehensively performed. Finally, additional investigations using patient-derived models and chemotherapy-resistant oral cancer cells would further strengthen the translational significance of these findings.

Collectively, the present study identifies quercetin as a selective multi-target anti-cancer compound capable of modulating apoptosis, autophagy, oxidative stress, DNA damage, metastatic signaling, and chemotherapy sensitivity in gingival squamous cell carcinoma. These findings support the translational potential of quercetin as a complementary therapeutic strategy in oral oncology and provide a strong rationale for future preclinical and clinical investigations.

## 5 Conclusion

Collectively, the present study demonstrates that quercetin exerts selective and potent anti-cancer activity against gingival squamous cell carcinoma through the coordinated modulation of multiple oncogenic pathways. Quercetin significantly inhibited cell proliferation, clonogenicity, migration, and metastatic signaling while promoting apoptosis and DNA damage in Ca9–22 cells. These effects were further associated with suppression of autophagy and oxidative stress, highlighting the multi-target nature of quercetin-mediated anti-tumor activity.

Importantly, quercetin displayed limited cytotoxicity toward non-malignant oral epithelial cells, supporting a potentially favorable therapeutic index. In addition, quercetin enhanced the anti-proliferative efficacy of cisplatin and 5-fluorouracil, suggesting its potential utility as a chemosensitizing agent in oral cancer therapy. Transcriptomic, survival, and computational analyses further supported the biological and translational relevance of apoptosis- and DNA damage-related pathways modulated by quercetin.

To our knowledge, this study is among the first to comprehensively demonstrate the selective vulnerability of gingival squamous carcinoma cells to quercetin through simultaneous targeting of apoptosis, autophagy, oxidative stress, DNA damage, and metastatic pathways. These findings support the translational potential of quercetin as a complementary therapeutic strategy in oral oncology. Nevertheless, additional *in vivo* studies and mechanistic investigations are required to validate these observations and to further assess the clinical applicability of quercetin in chemotherapy-sensitive and chemotherapy-resistant oral cancer models.

## Data Availability

The original contributions presented in the study are included in the article/supplementary material. Further inquiries can be directed to the corresponding author.
